# What are possible barriers and facilitators to implementation of a Participatory Ergonomics programme?

**DOI:** 10.1186/1748-5908-5-64

**Published:** 2010-08-24

**Authors:** Maurice T Driessen, Karin Groenewoud, Karin I Proper, Johannes R Anema, Paulien M Bongers, Allard J van der Beek

**Affiliations:** 1 Body@Work TNO VUmc, Research Center Physical Activity, Work and Health, VU University Medical Center, van der Boechorststraat 7, 1081 BT Amsterdam, The Netherlands; 2 Department of Public and Occupational Health, EMGO Institute for Health and Care Research, VU University Medical Center, van der Boechorststraat 7, 1081 BT Amsterdam, The Netherlands; 3 TNO Quality of Life, Polarisavenue 151, 2132 JJ, Hoofddorp, The Netherlands

## Abstract

**Background:**

Low back pain (LBP) and neck pain (NP) are common among workers. Participatory Ergonomics (PE) is used as an implementation strategy to prevent these symptoms. By following the steps of PE, working groups composed and prioritised ergonomic measures, and developed an implementation plan. Working group members were responsible to implement the ergonomic measures in their departments. Little is known about factors that hamper (barriers) or enhance (facilitators) the implementation of ergonomic measures. This study aimed to identify and understand the possible barriers and facilitators that were perceived during implementation.

**Methods:**

This study is embedded in a cluster randomised controlled trial that investigated the effectiveness of PE to prevent LBP and NP among workers. For the purpose of the current study, questionnaires were sent to 81 working group members. Their answers were used to make a first inventory of possible barriers and facilitators to implementation. Based on the questionnaire information, 15 semi-structured interviews were held to explore the barriers and facilitators in more detail. All interviews were audio taped, transcribed verbatim, and analysed according to a systematic approach.

**Results:**

All possible barriers and facilitators were obtained from questionnaire data, indicating that the semi-structured interviews did not yield information about new factors. Various barriers and facilitators were experienced. The presence of implementation plans for ergonomic measures that were already approved by the management facilitated implementation before the working group meeting. In these cases, PE served as a strategy to improve the implementation of the approved measures. Furthermore, the findings showed that the composition of a working group (*i.e.*, including decision makers and a worker who led the implementation process) was important. Moreover, stakeholder involvement and collaboration were reported to considerably improve implementation.

**Conclusions:**

This study showed that the working group as well as stakeholder involvement and collaboration were important facilitating factors. Moreover, PE was used as a strategy to improve the implementation of existing ergonomic measures. The results can be used to improve PE programmes, and thereby may contribute to the prevention of LBP and NP.

**Trial registration number:**

ISRCTN27472278

## Background

The lifetime prevalence rates of low back pain (LBP) and neck pain (NP) in western countries are high (90%), indicating that almost every person will experience an episode of LBP and NP during his/her life [1,2]. Furthermore, LBP and NP have considerable consequences for workers, companies, and society [3,4]. Therefore, preventing these symptoms at the workplace is imperative.

To prevent LBP and NP among workers, ergonomic measures are frequently implemented at the workplace. The findings of a recent systematic review, however, showed that the implementation of physical and organisational ergonomic interventions alone were not effective to prevent LBP and NP [5]. Therefore, the use of an adequate strategy to implement ergonomic measures, such as participatory ergonomics (PE), has been recommended. PE has already shown promising results in preventing of musculoskeletal disorders (MSD) [6]; however, the positive effects on MSD have not been confirmed by large randomised controlled trials (RCT) [7].

Another large cluster-RCT, the Stay@Work study, evaluated the effectiveness of a PE programme as an implementation strategy to prevent LBP and NP among workers [8]. As part of the PE programme, working groups had to implement ergonomic measures in their department. The process evaluation of this RCT has shown that one-third of the proposed ergonomic measures were implemented in the intervention departments [9]. From the literature it is known that various factors can positively or negatively influence implementation [10-12], including ergonomic measures derived from a PE programme [13,14]. Moreover, it has been postulated that factors for implementation can be present at different levels (*i.e.*, individual professional, worker, societal, or organisational) [15]. Knowledge on the barriers and facilitators about their presence in the different levels of the occupational context is crucial to improve the implementation of ergonomic interventions, thereby contributing to the reduction of LBP and NP among workers [16,17]. Nevertheless, the reporting on barriers and facilitators for implementation is lacking in most ergonomic intervention studies [18].

Therefore, embedded in a RCT, this study aimed to identify possible factors that hampered (barriers) and/or enhanced (facilitators) the implementation of the prioritised ergonomic measures when using the PE programme as an implementation strategy. It also aimed to understand how these barriers and/or facilitators influenced the implementation.

## Methods

More details on the methods of the Stay@Work PE programme, evaluation of the PE programme, and the perceived implementation have been published elsewhere [8,9]. The study protocol was approved by the Medical Ethics Committee of the VU University Medical Center.

### Study setting and intervention

Stay@Work was designed as a cluster-RCT to investigate the effects of a PE programme to prevent LBP and NP among workers. Based on their workload, 37 departments from four Dutch companies (a railway transportation company, an airline company, a university including its university medical hospital, and a steel company) were classified into: mentally, mixed mentally and physically, light physically, or heavy physically demanding work [19]. To avoid contamination from workers allocated in the intervention group to those in the control group randomisation was performed at a departmental level. Within each company, pairs of departments with comparable workloads were randomly allocated to either the PE intervention group or the control group (no PE). By using a computer-generated randomisation programme, 19 departments were allocated to the intervention group and 18 to the control group.

Each intervention department formed a working group, consisting of eight workers and one (department) manager. Workers invited for the working group had to have worked at least two years in their current job, and for more than 20 hours per week in the department. The (department) manager in the working group, had to have decision authority on organisational and financial aspects.

Under the guidance of an ergonomist, 16 working groups (for 19 intervention departments) followed the steps of the Stay@Work PE programme during a six-hour working group meeting. In this meeting, working group members added risk factors of LBP and NP, and judged all mentioned risk factors on their frequency and severity (step one). Based on the perceptions of the working group, the most frequent and severe risk factors were prioritised, resulting in a top three of risk factors (step two). Subsequently, the working group held a brainstorming session about different types of ergonomic measures to target the prioritised risk factors and evaluated the ergonomic measures according to a criteria list considering: relative advantage, costs, compatibility, complexity, triability, feasibility, and visibility [20]. Further, the ergonomic measures had to be implementable within a timeframe of three months. On a consensus basis, the working group prioritised the three most appropriate ergonomic measures (step three). An implementation plan was formed containing information on the prioritised risk factors for the development of LBP and NP and the prioritised ergonomic measures to prevent LBP and NP (step four). The implementation plan also described which working group member(s) was/were responsible for the implementation of the prioritised ergonomic measure(s); these working group members were called 'implementers.' At the end of the meeting, the working group was requested to implement the ergonomic measures (step five) and was asked whether an appointment for a second, optional meeting was necessary to evaluate or adjust the implementation process (step six). Altogether the working group meetings resulted in 66 prioritised ergonomic measures. According to the classification by van Dieën and van der Beek (2009) the prioritised ergonomic measures were classified into three categories [21]: individual ergonomic measures that were aimed at the individual worker (*i.e.*, improving awareness regarding ergonomics, worksite visit, physical activity programs); physical ergonomic measures that were aimed at redesigning the workplace (*i.e.*, ergonomic modification, new equipment, or manual handling aids), and organisational ergonomic measures that were aimed at changing the system level (*i.e.*, pause software installation, job rotation, or restructuring management style). Most of the prioritised ergonomic measures addressed either individual (n = 32) or physical (n = 27) ergonomic measures, whereas organisational ergonomic measures (n = 7) were less prevalent [9]. To improve the implementation process, two or three implementers from each working group were asked to voluntary follow a training programme to become a Stay@Work ergocoach. A total of 40 implementers attended the ergocoach training [9]. In this additional four-hour implementation training, they were educated in different implementation strategies to inform, motivate, and instruct their co-workers about ergonomic measures. Moreover, ergocoaches were equipped with a toolkit consisting of flyers, posters, and presentation formats. These types of implementation strategies have been recommended to induce behavioural change [22,23].

### Data collection and analyses

Data were collected from the so-called 'implementers,' who were working group members responsible for the implementation of one or more prioritised ergonomic measure(s).

### Questionnaires

To identify barriers and facilitators to implementation, all implementers (n = 81) received a questionnaire four months after finishing the first working group session. By means of open questioning, the implementers were asked to report on the perceived barriers and/or facilitators to those ergonomic measures he/she was responsible for. To assist the implementers, researchers provided several examples of barriers in the questionnaire. Furthermore, to understand 'how' the barriers and facilitators influenced implementation, the implementers were asked to provide a brief explanation for each barrier or facilitator. A total of 65 implementers (80%) responded on the questionnaire. Among the responders were 35 males (54%) and 30 females (46%); 52 of the responders (80%) were workers, whereas 13 had a management function (20%). Moreover, most responders worked in a department characterised by either a mental workload (42%) or a heavy physical (30%) workload (see Table 1).

**Table 1:** Characteristics of the participating implementers

	Questionnaire responders (n = 65)	Interviewed implementers (n = 15)
Male/Female	35/30	8/7

Worker/Manager	52/13	8/7

Heavy physical demanding work	20	2

Light physical demanding work	4	2

Mental demanding work	27	6

Mix mental/physical demanding work	14	5

### Questionnaire data analyses

First, an inventory of possible barriers and facilitators for each working group was made. This was performed by two researchers (MTD and KG), who independently extracted all possible barriers and facilitators for implementation from the questionnaires. During a consensus meeting, the two researchers discussed whether all possible barriers and facilitators were obtained.

Based on the inventory, the semi-structured interviews were developed to explore the barriers and facilitators in further detail, and potential participants for the interviews were selected.

### Semi-structured interviews

The aim of the semi-structured interview was to: verify the correctness of barriers and facilitators derived from the questionnaires; gain in-depth understanding as to 'how' the barriers and facilitators influenced implementation; and gather new barriers and facilitators. The interview was held only among implementers from those working groups that had finished the implementation period (n = 9 working groups). To acquire a broad overview of implementation factors, from each working group we intended to interview one implementer who participated as a manager and one implementer who participated as a worker. Moreover, we tried to select implementers who fulfilled a key role in the implementation process of their working group (*i.e.*, had to implement most of the prioritised ergonomic measures). Furthermore, we intended to select the implementers from different departments (*i.e.*, mental or heavy physical) and different companies (see Table 1).

Potential participants for the semi-structured interview were selected among the implementers who responded to the questionnaire. Implementers were contacted by the principal researcher (MTD) by telephone and were invited to a face-to-face interview. One week before the start of the interview, the implementer was emailed an overview of the perceived barriers and facilitators (with explanation) that were reported by the other implementers from his/her working group. During the interview a guide was used to ensure that the same semi-structured questions were addressed. All interviews were conducted by the principal researcher and took place in person with only the researcher and the implementer present. The interview had a mean duration of 30 minutes, and all interviews were recorded on a digital voice recorder. No more than two interviews were held on the same day. All interviewed implementers provided informed consent.

### Semi-structured interview data analyses

First, all interviews were transcribed verbatim. Two researchers (MTD and KG) independently extracted all possible barriers and facilitators to implementation from the transcripts. Data extracted from the transcription sets was subsequently analysed using the constant comparison process [24,25]. By following this process, the two researchers independently checked whether all possible barriers and facilitators that were obtained from the questionnaires were also obtained from the semi-structured interviews. Moreover, it was checked whether new barriers and facilitators were derived from the semi-structured interviews. To ensure uniformity on the identified barriers and facilitators, a consensus meeting between the two authors was held. For all data extracted, a qualitative software program (Atlas.ti version 5.2) was used to electronically code and manage data, and to generate reports of coded text for analysis. To illustrate the meaning of the perceived barriers and facilitators, quotations that were considered representative for each barrier or facilitator were reported in the text. Quotations were derived from the semi-structured interviews and were translated from Dutch.

### Classification of perceived barriers and facilitators into implementation levels

After reaching consensus on the barriers and facilitators for implementation obtained from the questionnaires and the semi-structured interviews, the researchers (MTD and KG) classified the perceived barriers and facilitators into different implementation levels by using the 'implementation model' of Grol and Wensing (2004) [15]. By classifying the implementation factors into implementation levels more specific recommendations to improve implementation can be formulated. The model was originally used in the healthcare setting and distinguished six implementation levels in which barriers and facilitators for implementing an innovation could be perceived: the innovation itself (*i.e.*, feasibility, accessibility, and advantages in practice); the individual professional (*i.e.*, awareness, motivation to change, and routines); the patient (*i.e.*, knowledge, skills, and attitude); the social context (*i.e.*, culture of network, opinions of colleagues, and leadership); organisational (*i.e.*, staff, capacities, and resources); and economical and political context (*i.e.*, regulations, policies, and financial arrangements) [15].

## Results

All barriers and facilitators were derived from the questionnaire data; that is, the interviews did not yield any additional barriers or facilitators. Table 2 presents the perceived barriers and facilitators from the perspective of the implementers and stratified for the four implementation levels. Because the original implementation levels used by Grol and Wensing (2004) were based on the healthcare setting, some of the levels were not applicable to the workplace in which our study was conducted. Adjustments were made to create more context-specific levels. The 'economic and political context,' 'patient,' and 'individual professional' levels were excluded because no barriers and facilitators were identified on these levels. In the model by Grol, the social context is a rather wide perspective including the culture and existing values of the network, perceived patients expectations and behaviour, and collaboration between healthcare teams. In the current study, the social context encompassed only the implementers' co-workers, and therefore the 'social context' was replaced by a co-worker level. The working group level was introduced because the working group itself is a specific characteristic of a PE programme, and referred to the barriers and facilitators perceived by the implementers at the level of the working group. Because in the current study the innovations encompassed the implementation of ergonomic measures, the term 'innovation' was replaced by an ergonomic measure level.

**Table 2:** Perceived barriers and facilitators to implementation by the implementers

Implementation level	Factor	Explanation(s) of factors
Organisational	Management commitment	- (No) agreement or (no) support from management to implement prioritised ergonomic measure (**b+f**)
	
	Resources	- (Lack of) financial resources **(b+f) **- (Lack of) personnel resources (**b+f**)
	
	Collaboration	- Implementation process was delayed or accelerated by persons/structures/services within or outside the department (**b+f**)

Co-worker	Culture	- Prioritised ergonomic measure did not fit in the department culture (**b**)

Working group	Composition	- (No) leading person in the working group (**b+f**) - Members dropped out from or stayed in the working group (**b+f**) - Members had (no) time for implementation (**b+f**) - No decision maker in working group (**b**) - Efforts made by working group members (**f**)

Ergonomic measure	Relative Advantage	- Prioritised ergonomic measure did (not) improve the situation when compared to the current situation (**b+f**)
	
	Difficulty	- Prioritised ergonomic measure were easy/difficult to implement (**b+f**)
	
	Compatibility	- Prioritised ergonomic measure did not fit the workplace (**b**)
	
	Complexity	- Prioritised ergonomic measure was not direct practicable for all workers (**b**)
	
	Approved	- The plans for implementing the prioritised ergonomic measure were already made and approved before the working group meeting took place (**f**)

b + f: explanation could be both a barrier and a facilitator

b: explanation of a barrier

f: explanation of a facilitator

Table 2 presents the explanations of the perceived barriers and facilitators to implementation. While some factors were perceived as either a barrier or facilitator, most of the factors were experienced as being both a barrier and a facilitator. Most factors (n = 5) for implementation were found at the level of the ergonomic measure.

### Organisational level

At the organisational level, three factors appeared to be perceived as both a barrier and facilitator. The three factors were 'management commitment,' 'resources,' and 'collaboration.'

#### Management commitment

The factor 'management commitment' referred to whether the management supported or did not support the implementation of the prioritised ergonomic measure. Despite a (department) manager or its representative attending the working group meeting and approving the implementation of the prioritised ergonomic measure, the implementers still reported this factor as being important for implementation. Management commitment was in most cases mentioned as a facilitator. During the interview one of the implementers said:

'There were, of course, the managers at the department but they were fine with it [the prioritised ergonomic measure] and supported the initiative to be more aware on work and health. They [the managers] were happy with it. So from that point everybody was enthusiastic!'

#### Resources

At the organisational level, the factor 'resources' had two meanings. Most frequently, implementers reported that implementation was hampered due to insufficient financial resources. Insufficient financial resources most often played a role during the implementation of physical ergonomic measures (*i.e.*, new chairs). During the interview one implementer explained the financial resources as:

'Our management reserved an implementation budget to implement the new chairs.'Other implementers mentioned that it was a lack of personnel resources that hampered implementation. This problem most often occurred when organisational ergonomic measures such as job rotation had to be implemented. Regarding the personnel resources implementers said:

'There are many practical factors which make it impossible to do something with this ergonomic measure. At this moment this is mainly caused by the enormous lack of personnel resources.'

#### Collaboration

The factor 'collaboration' referred to the collaboration with persons, structures, or services within or outside the department during the implementation process, and was mostly experienced as a barrier. Implementers blamed the bureaucracy of their firm or their own department, and reported that key persons for implementation (*i.e.*, engineers, technicians, or suppliers) or other services (*i.e.*, equipment or health services) were too busy to help them with implementing the ergonomic measures. Other implementers had positive experiences with collaboration and reported that collaboration facilitated the implementation of the ergonomic measure. One of the implementers said:

'We received good help [from two persons of the occupational health services]. They knew our department very well, and very soon we had all information for our training available.'

### Co-worker level

#### Culture

At the level of the co-worker, only the implementation factor 'culture' was identified. The factor 'culture' referred to which extent the prioritised ergonomic measure fit within the culture of the department. One implementer reported that the reactions and opinions of some co-workers were so negative that he decided to stop with the implementation of the ergonomic measure. During the interview he said:

'So, drawing attention to each other's working posture [the prioritised ergonomic measure] is not really incorporated into our department culture. They [the co-workers] find that annoying and it bothers them. The same goes for the managers. Sometimes they [the co-workers] say things to me like: 'what is your problem?' or 'leave it, it's my body!' So, that's why I stopped doing it.'

### Working group level

#### Composition

At the level of the working group, the only factor for implementation that was identified was 'composition' and was experienced by many implementers in different working groups. The factor was experienced as both a barrier and a facilitator, and can have different explanations.

According to many implementers, 'composition' was facilitating if there was one implementer in the working group who played a leading role during the implementation process, while not having such a leader was experienced as a barrier. During the interview one implementer said:

'In my opinion this is because she spent all her efforts on the implementation and if she wants something then it has to be done. She doesn't stop before she's reached her goal, and that was a really important factor for this measure.'

With special emphasis towards the implementation of individual ergonomic measures, implementers from departments characterised by a mental workload reported that 'composition' hampered implementation because of the high number of dropouts in their working group. As a consequence, too few persons were left in the working group to implement all prioritised ergonomic measures.

Some implementers had too many other work-related tasks and thereby lacked the time to play an active role in the implementation process. Others reported that 'composition' hampered implementation, because their working group lacked a person who was entitled to make decisions at departmental level. Consequently, the decisions had to be approved by another (higher) management level.

### Ergonomic measure level

The following factors for implementation were reported at the level of the ergonomic measure: 'relative advantage,' 'difficulty,' 'compatibility,' 'complexity,' and 'approved.'

#### Relative advantage

The factor 'relative advantage' was defined as the possible effects that the ergonomic measure could have in terms of LBP and NP prevention among workers at the department compared to the current situation. According to some implementers, this factor was a facilitator if during the implementation they remained convinced of the relative advantage of the prioritised ergonomic measure. However, with special regard to physical ergonomic measures, most implementers reported that during the implementation they discovered that the relative advantage of the prioritised ergonomic measure was little compared to the current situation. In these cases, little relative advantage was perceived as a barrier. One of the implementers said during the interview:

'We thought that five patients a day would be transferred by using this lifting device [the prioritised ergonomic measure], however, in practice this is not true [more than five patients]. OK, the lifting device costs some money but that is not the problem, the most important point is its advantage. Regarding its advantage, I'm still not convinced.'

#### Difficulty

The factor 'difficulty' was defined as to the extent to which the ergonomic measure was difficult to implement. Some implementers reported that implementation was hampered because the ergonomic measures were too difficult to implement within three months. Most implementers experienced easy implementations as a facilitator:

'It was a really simple task, and yes that was important. Some things you just have to do quickly and I think that these quick successes are important.'

#### Compatibility

The factor 'compatibility' referred to the extent to which the ergonomic measure was compatible with the present norms and practises in the department. In other words, how well the innovation 'fit' into the department. Compatibility is positively related to the rate of implementation. However, in this study a few implementers reported that the prioritised ergonomic measure was not very compatible at the department and implementation was hampered. One of these implementers said:

'I collected information on this, but it [screensaver with ergonomic advices] was not compatible on the computers, so it could not be implemented. That was to my opinion a technical problem.'

#### Complexity

The factor 'complexity' referred to the extent to which the workers were able to understand and use the ergonomic measure after it had been implemented. Less complex ergonomic measures are positively related to the rate of implementation. Nevertheless, in this study 'complexity' was only perceived as a barrier when the ergonomic measure appeared to be too complex for the workers to immediately understand and to use it. During the interview one of the implementers said:

'In addition, if we would have implemented the carts, workers had to follow special training sessions on how to use them.'

#### Approved

The factor 'approved' referred to the extent to which plans for implementing the ergonomic measure were already present and approved by the (department) management before the working group meeting was held. Many implementers of different working groups mentioned that this was the case for some of the ergonomic measures they prioritised and experienced that this facilitated the implementation process. One of the implementers said:

'Well, the plans to implement new chairs were already made, even before the working group meeting was held. So, when the working group prioritised to implement the new chairs, it was not so difficult to order them.'

## Discussion

The aim of this study was to identify possible factors that hampered or facilitated the implementation of the prioritised ergonomic measures that were derived from a PE programme. The findings of this study suggested that various barriers and facilitators to implementation were perceived at four implementation levels. Insight into the barriers and facilitators to implementation is useful, because it shows what kind of (sometimes unforeseen) factors may occur when implementing ergonomic measures. Moreover, the results may contribute towards the improvement of PE programmes as an implementation strategy. As a consequence of improved implementation, LBP and NP among workers may be reduced.

### Comparison with other studies

Previous studies have reported on the barriers and facilitators that were experienced during a PE programme. For example, the PE framework by Haines *et al. *(2002) described important implementation dimensions (*i.e.*, level of influence of the working group, guiding role of ergonomist, and direct involvement of workers) that should be considered during the development a PE programme [26]. Moreover, a systematic review by van Eerd and colleagues (2008) identified barriers and facilitators for the process and implementation of a PE programme and classified them into 19 categories (*e.g.*, resource availability, creation of an appropriate team, and sufficient resources) [27]. Many similarities were found when comparing our main findings with the study findings of Haines *et al. *(2002) and van Eerd *et al. *(2008) [26,27]. It was found that almost the same definitions were used to point out the meaning of the barriers and facilitators. However, due to the use of a different framework or model, the labelling of the barriers and facilitators slightly differed between the studies. For example, Haines *et al. *(2002) used the label 'mix of participants' to address the importance of incorporating a mixed group of participants in the working group (*i.e.*, operators, supervisors, technical staff, and management) while we named this 'composition' at the working group level. Furthermore, the implementation levels or dimensions that were used to classify barriers and facilitators differed between studies. Because our study aim was to identify all possible barriers and facilitators on implementation, we used the implementation model by Grol and Wensing (2004) in which not only contextual levels were incorporated but also the level of the ergonomic measure was considered.

Our findings were in concordance with the results of other PE studies that used qualitative research methods. Factors that hamper implementation have included high production pressures, not securing employees' time to carry out ergonomic changes, lack of management commitment, insufficient financial resources, and workers' frustration due to implementation delays [13,14,28].

Although most of the barriers and facilitators obtained from other PE studies were in line with our findings, caution is needed when comparing the results. This is because heterogeneity existed regarding the study design, study population, outcome measures, type of ergonomic changes, the timing, and methods used to assess barriers and facilitators for implementation (mix of questionnaires and semi-structured interviews).

### Implications

The findings of this study offered new information on factors to implementation of ergonomic measures using the PE implementation strategy. It appeared that implementation was facilitated if plans for implementing the ergonomic measure were already present and were approved by the management before the working group meeting took place. This may indicate that the PE implementation strategy can not only be used to develop new ergonomic measures, but also to improve the implementation of the already planned ergonomic measures in a department. This finding is not surprising because it is known that most ergonomic measures are implemented without using an adequate implementation strategy [29]. Despite all of the prioritised ergonomic measures meeting the implementation criteria (*i.e.*, low initial costs and less complex, large relative advantage, compatible, good triability, visible, and feasible) [20], our findings show that meeting these criteria alone does not guarantee implementation. With special regard to physical ergonomic measures, some implementers discovered during the implementation process that it was too costly to order the measure for the whole department and consequently the implementation was reconsidered. To avoid these types of problems, we included a manager in the working group who had sufficient decision authority to facilitate implementation. However, this seemed not to be sufficient. Our findings show that the involvement of stakeholders may improve implementation since these professionals have more knowledge on the costs and/or the working mechanisms of ergonomic measures. Therefore, incorporating important stakeholders (such as technicians, engineers, suppliers, or occupational health experts) into the working group or consulting them during the implementation process is recommended [30]. Furthermore, we found that it was important to create an enthusiastic and sustainable working group that is supported by its management and supplied with sufficient resources (*i.e.*, time and money).

### Strengths and limitations

The factors for implementation were obtained from a heterogeneous working population; therefore, the findings represent a broad overview of possible barriers and facilitators. Furthermore, few studies on the factors for implementation of ergonomic interventions have used qualitative research methods [31]. The use of qualitative research techniques can result in a better understanding of the meaning of the factors for implementation [24]. Further strengths of this study were that data were analysed using a systematic approach [24,25] and an adapted version of the well-known theoretical implementation model by Grol and Wensing (2004) was used to classify the barriers and facilitators into levels [15].

However, there were also some limitations in our study. A selected group of implementers was interviewed--only implementers from working groups that had finished the full implementation period. The selection of this group of implementers may have influenced the representativeness of this study. We do not believe that this selection resulted in less communication of barriers, because all barriers and facilitators were derived from the questionnaire data. Bias may have occurred because the interviews were conducted by the principal researcher. Moreover, implementers knew the researcher and were familiar with the position of the researcher in the research project [32], which could have sometimes resulted in 'socially accepted answers.' Another limitation is that the barriers and facilitators were obtained from the implementers' point of view, whereas other persons from different levels (*i.e.*, management, health services, or co-workers) were involved during the implementation as well. It would be informative to gain insight into which barriers and facilitators to implementation these persons experienced.

## Summary

In summary, the findings show that PE can be used for both the development and implementation of new ergonomic measures as well as to improve implementation of already planned ergonomic measures. Furthermore, the working group composition was important for implementation, meaning that a manager who is entitled to make decisions at the department level and working group members who can play a leading role during the implementation process should be included. Stakeholder involvement can considerably facilitate implementation; therefore, it is recommended that they are involved in the working group or consulted during the implementation process. The results of this study can be used to further improve PE programmes as a strategy for implementation. As a consequence of improved implementation, LBP and NP prevalence among workers may be reduced.

## Competing interests

The authors declare that they have no competing interests.

## Authors' contributions

All authors contributed to the design of the study. MTD is the principle researcher and was responsible for the data collection and data analyses. KG conducted the data analyses. KIP, JRA, PMB, and AJvdB supervised the study. All authors contributed to the writing up of this paper and approved the final manuscript.
